# Evaluation of Serum Levels of Insulin-Like Growth Factor 1 and Insulin-Like Growth Factor-Binding Protein 3 in Patients With Colorectal Cancer: A Case-Control Study

**DOI:** 10.7759/cureus.19881

**Published:** 2021-11-25

**Authors:** Rania Naguib, Mohamed Abouegylah, Sherif Sharkawy, Amel A Fayed, Hend Naguib

**Affiliations:** 1 Clinical Science Department, College of Medicine, Princess Nourah Bint Abdulrahman University, Riyadh, SAU; 2 Internal Medicine Department, Endocrinology Unit, Alexandria Faculty of Medicine, Alexandria, EGY; 3 Department of Clinical Oncology, Faculty of Medicine, Alexandria University, Alexandria, EGY; 4 Clinical Pathology Department, Faculty of Medicine, Alexandria University, Alexandria, EGY; 5 Internal Medicine Department, Hepatology Unit, Faculty of Medicine, Alexandria University, Alexandria, EGY

**Keywords:** clinicopathological features, colorectal cancer, insulin-like growth factor binding protein 3, insulin-like growth factor1, case control studies

## Abstract

Background/aim: Limited information is available about the relationship between colorectal cancer (CRC) and serum concentration of insulin-like growth factor 1 (IGF1) and insulin-like growth factor-binding protein 3 (IGFBP3). This study aims to compare the serum levels of IGF1and IGFBP3 in colorectal cancer cases and controls and to assess the relationship between their level and the demographic and histopathological characteristics.

Methods: A case-control study in which 50 patients with colorectal cancer and 50 controls matched by gender and age were compared regarding the demographic characteristics and the level of both IGF1 and IGFBP3. The correlation with different clinicopathological features was assessed.

Results: Levels of IGF1 were significantly higher while levels of IGFBP3 were significantly lower among cases compared to control. IGF1 was significantly higher among patients with liver metastasis, lymph node (LN) spread, and lymphovenous invasions and did not show significant association with gender, smoking status, family history, or primary site of colorectal cancer. Lower IGFBP3 was significantly high among patients with liver and lymph node metastasis, lymphovenous invasion, and patients with positive family history. This significant negative correlation was also detected between IGFBP3 levels and the size of the tumor.

Conclusions: High IGF1 levels and low concentrations of IGFBP3 are related to colorectal cancer and were significantly associated with liver metastasis, lymph node spread, and lymphovenous invasions. Further research is recommended to investigate if circulating IGF1 and IGFBP3 levels can be used to identify people at high risk of colorectal cancer and to investigate potential lifestyle or pharmaceutical ways to lower IGF1 bioactivity as a risk reduction strategy.

## Introduction

Colorectal cancer (CRC) is the third most prevalent cancer worldwide and the second most common cancer-causing mortality [1,2]. The number of cases is expected to go up by 60% reaching up to more than 2.2 million new cases and 1.1 million deaths by the year 2030 [2,3]. A relationship between various components of the insulin-like growth factor system (IGFs) and the development of cancers, both solid tumors and hematological malignancies, has been noted for several decades [3-6].

Two ligands (IGF1 and IGF2), two cell-surface receptors (IGF1R and IGF2R), a family of six high-affinity IGF binding proteins (IGFBPs 1 to 6), and four additional low-affinity binding proteins make up the IGFs. The IGFBPs have a high affinity for an insulin-like growth factor (IGF), and their primary purpose is to monitor the availability of IGF for interactions with IGFR. IGF-binding protein3 (IGFBP3) has been a focus of different studies since it is the most abundant binding protein in the circulation accounting for nearly 80% of all IGFBPs [7-9]. Approximately 98% of IGF1 in the serum is inactive due to binding to the IGFBPs. The IGFs are involved in normal cell growth, neoplastic transformation, tumor production, and metastasis. In several malignancies, including CRC, abnormal expression of IGF, as well as their receptors and binding proteins, have been reported [3,4,10,11].

IGF1 is involved in a number of carcinogenesis-related processes, including cell proliferation and apoptosis leading to an increased risk of cancers [7-9,12]. Higher cancer risk was reported to be associated with acromegaly, which is characterized by increased growth hormone levels with concomitant elevated levels of IGF1 [13]. Furthermore, recent observational studies reported that IGF1 levels have been shown to be elevated in non-acromegalic populations with colon cancer. Elevated serum IGF1 levels have been associated with an increased risk of CRC, prostate, and breast cancer [14]. The elevated serum levels of IGF1 in CRC make it possible to identify the components of the IGF system as biomarkers of human CRC predisposition and prognosis [3,8,15]. IGFBP3 has been shown to exhibit direct antiproliferative and pro-apoptotic effects [14]. Low IGFBP3 levels in the blood have been related to an increased risk of cancer [6]. This case-control study was conducted to compare the serum levels of IGF1 and IGFBP3 in CRC cases and control subjects and to assess the relationship between their level and the demographic and histopathological characteristics of CRC.

## Materials and methods

Study design

This is a case-control study that was conducted during the period between February and July 2021. The study was approved by the Institutional Ethics and Research Committee at Alexandria Faculty of Medicine (IRB approval number 21-227). Written informed consent was taken from all participants.

Inclusion and exclusion criteria

A total of 50 patients with CRC were recruited from Alexandria Main University Hospital, Oncology, Surgery, and Internal Medicine Departments, outpatient clinics. Fifty healthy subjects with matched age and sex who do not have any clinical manifestations of CRC and have negative stool analysis for occult blood were used as control. The inclusion criteria were age at least 18 years. Exclusion criteria included those who reported oral contraceptive and hormone replacement therapy at recruitment (as oral estrogens exert a strong first-pass effect on the liver that alters hepatic protein production and changes circulating levels of multiple hormones, including IGF-system hormones); those who reported type-2 diabetes or unknown diabetes status at recruitment (as diabetes medications can affect circulating levels of IGF proteins) [14]. None of the patients had received chemotherapy or radiotherapy or had any other malignancies. Diagnosis of CRC was based on a pathology report. The staging of CRC was based on CT findings.

Data collection

Demographic data of the patients including age, sex, family history, and smoking history were reported. The height of all participants was measured at baseline by trained staff using standardized protocols and was recorded in centimeters. Histopathological characteristics of the tumor such as the anatomical site (proximal colon, distal colon, and rectal cancer), size, histological type, presence of liver metastasis, presence of lymph node (LN) metastasis, and presence of lymphovascular invasion were documented. Proximal colon cancers included those found in the caecum, appendix, ascending colon, hepatic flexure, transverse colon, and splenic flexure while descending and sigmoid colon cancers were considered as distal colon tumors and tumors in recto-sigmoid junction and rectum were considered as rectal cancer [16].

Measurements of IGF1 and IGFBP3

Peripheral blood samples were drawn in serum separator tubes. After sampling, tubes were centrifuged at 2000 *g* for five minutes. The obtained serum for each subject was aliquoted and stored at −20 °C till analysis. IGF1 and IGFBP3 concentrations were measured using respective Quantikine™ enzyme-linked immunosorbent assay (ELISA) kits (R&D Systems, Inc., Minneapolis, MN) following the manufacturer's instructions.

Statistical analysis

Patients and control subjects were compared regarding the demographic characteristics and the level of both IGF1 and IGFBP3 and the correlation with different clinicopathological features was assessed. Data entry and analysis were done using IBM SPSS software package version 20.0. (Armonk, NY: IBM Corp). The Kolmogorov-Smirnov test was used to verify the normal distribution of variables. Qualitative variables were described as frequencies and percentages, while normally distributed quantitative variables were described as means and SD. Comparisons between groups for categorical variables were assessed using the Chi-square test. Student t-test/analysis of variance (ANOVA) was used to compare two groups for normally distributed quantitative variables. Pearson correlation coefficient was used to test the correlation between numeric variables. A P-value less than 0.05 was considered statistically significant.

## Results

Cases and controls were matched according to age and gender distribution with comparable height and smoking habits (p-value > 0.05), whilst positive family history was reported by five cases (four cases in first degree relatives and one in second-degree relatives) compared to none by the controls (p-value 0.056). The average IGF1 was significantly higher among cases compared to the control group (231.8 ± 64.6 versus 129 ± 12.3; p-value < 0.001), whereas IGFBP3 was significantly lower among cases (2438.4 ± 855.4) than controls (3009.1 ± 441.3; p-value <0.001; Table 1).

**Table 1:** Comparison between the two studied groups according to different parameters

	Cases (n = 50)	Control (n = 50)	p-Value
Sex			
Male	30 (60%)	31 (62%)	0.838
Female	20 (40%)	19 (38%)	
Age (years)			
Mean ± SD	61.7 ± 9.9	61.8 ± 8	0.929
Median (min.–max.)	62 (43–79)	63 (43–77)	
Height (cm)			
Mean ± SD.	163.8 ± 4.2	162.6 ± 5.2	0.199
Median (min.–max.)	163 (156–174)	162.5 (154–174)	
Smoking			
No smoker	33 (66%)	28 (56%)	0.349
Smoker	8 (16%)	14 (28%)	
Ex-smoker	9 (18%)	8 (16%)	
Family history			
No	45 (90%)	50 (100%)	^FE^p=0.056
Yes	5 (10%)	0 (0%)	
IGF1 (ng/mL)			
Mean ± SD	231.8 ± 64.6	129 ± 12.3	<0.001^*^
Median (min.–max.)	223 (87–378)	127.5 (108–160)	
IGFBP3 (ng/mL)			
Mean ± SD	2438.4 ± 855.4	3009.1 ± 441.3	<0.001^*^
Median (min.–max.)	2655.5 (1126–4883)	2942.5 (2348–4017)	

Histopathological characteristics of the studied cases are displayed in Table 2. The majority of cases were of adenocarcinoma type (n=48, 96%) and mainly at the rectal region (44%). More than one-third of the patients had liver metastasis (38%) while nearly half of them had lymph node metastasis (46%) and lymphovascular invasion (52%).

**Table 2:** Anatomical and histopathological characteristics of colorectal cancer in a sample of 50 patients

	No. (%)
Site	
Distal	14 (28%)
Proximal	14 (28%)
Rectal cancer	22 (44%)
Size (cm)	
Mean ± SD	4.1 ± 2
Median (min.–max.)	3.8 (1.5–8.5)
Histological type	
Adenocarcinoma	48 (96%)
Adenosquamous carcinoma	1 (2%)
Undifferentiated carcinoma	1 (2%)
Liver metastasis	19 (38%)
LN metastasis	23 (46%)
Lymphovenous invasion	26 (52%)

IGF1 was significantly higher among patients with liver metastasis, lymph node spread, and lymphovascular invasions and did not show significant association with gender, smoking status, family history, or primary site of CRC. Nevertheless, lower IGFBP3 was significantly evident among patients with liver and lymph node metastasis and those with lymphovascular invasion in addition to patients with negative family history (Table 3).

**Table 3:** Anatomical and histopathological characteristics of colorectal cancer in a sample of 50 patients

	No.	IGF1 (ng/mL)			IGFBP3 (ng/mL)		
		Mean ± SD.	Median (min.–max.)	Test of sig. (p)	Mean ± SD	Median (min.–max.)	Test of sig. (p)
Sex							
Male	30	236.5 ± 65.7	223 (153–378)	t=0.626 (0.534)	2374.4 ± 835.2	2654 (1126–4232)	t=0.644 (0.523)
Female	20	224.8 ± 63.9	214 (87–347)		2534.4 ± 897.8	2791 (1354–4883)	
Smoking							
Non-smoker	33	224.1 ± 64.9	198 (87–370)	F=0.910 (0.410)	2624.4 ± 814.5	2830 (1126–4883)	F=2.903 (0.065)
Smoker	8	257.8 ± 75.2	258 (153–378)		2273.9 ± 995.4	2052 (1327–4232)	
Ex-smoker	9	237.1 ± 53.1	238 (165–314)		1902.7 ± 684.3	1537 (1254–2791)	
Family history							
No	45	228 ± 62.3	216 (87–370)	t=1.240 (0.221)	2323.2 ± 812.1	2615 (1126–4883)	t=3.099^*^ (0.003^*^)
Yes	5	265.6 ± 82.1	230 (196–378)		3475.6 ± 463.7	3279 (3105–4232)	
Site							
Distal	14	261.9 ± 91.7	280 (87–378)	F=2.231 (0.119)	2611.8 ± 884.3	2867 (1126–4232)	F=0.825 (0.444)
Proximal	14	221.8 ± 46.5	219 (156–289)		2204.9 ± 809	2345 (1254–3598)	
Rectal cancer	22	219 ± 48.4	207 (153–326)		2476.7 ± 871.8	2655.5(1257–4883)	
Liver Mets							
No	31	214.4 ± 72.1	193 (87–378)	t=2.979^*^ (0.005^*^)	2962.7 ± 593.7	2899 (1398–4883)	t=8.953^*^ (<0.001^*^)
Yes	19	260.2 ± 36.2	263 (165–318)		1583 ± 398.2	1432 (1126–2561)	
LN metastasis							
No	27	199.6 ± 59.6	193 (87–378)	t=4.506^*^ (<0.001^*^)	2889.9 ± 697.9	2835 (1398–4883)	t=4.902^*^ (<0.001^*^)
Yes	23	269.6 ± 48.2	265 (183–348)		1908.5 ± 714.3	1537 (1126–3354)	
Lymphovenous invasion							
No	24	197.7 ± 61.4	191.5 (87–378)	t=4.140^*^ (<0.001^*^)	3010 ± 621.4	2895.5(1398–4883)	t=5.903^*^ (<0.001^*^)
Yes	26	263.3 ± 50.6	264 (165–348)		1910.8 ± 689.6	1540 (1126–3354)	

**Figure 1:**
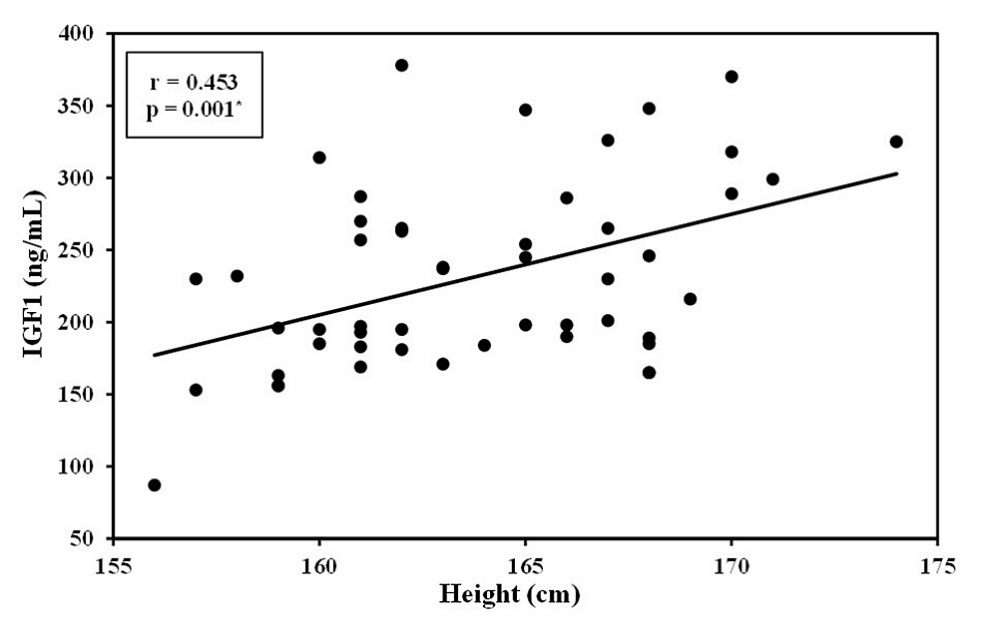
Correlation between IGF1 with height in case groups (n = 50) IGF1: insulin-like growth factor 1, r: correlation coefficient, p: probability

**Figure 2:**
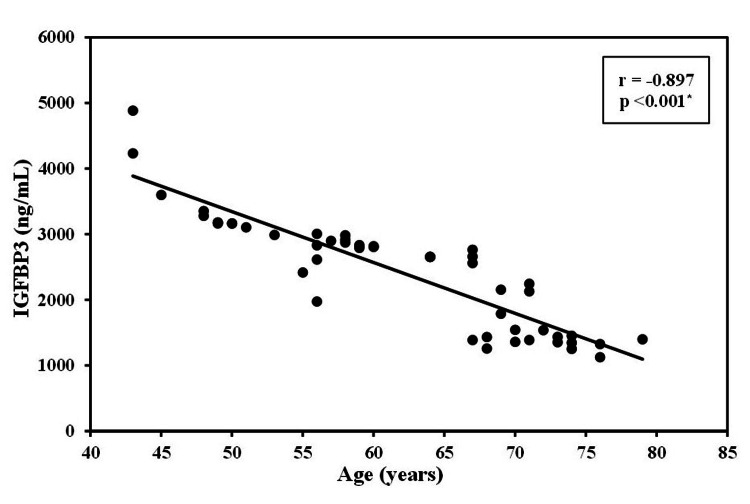
Correlation between IGFBP3 with age in case groups (n = 50) IGFBP3: insulin-like growth factor binding protein 3, r: correlation coefficient, p: probability

**Figure 3:**
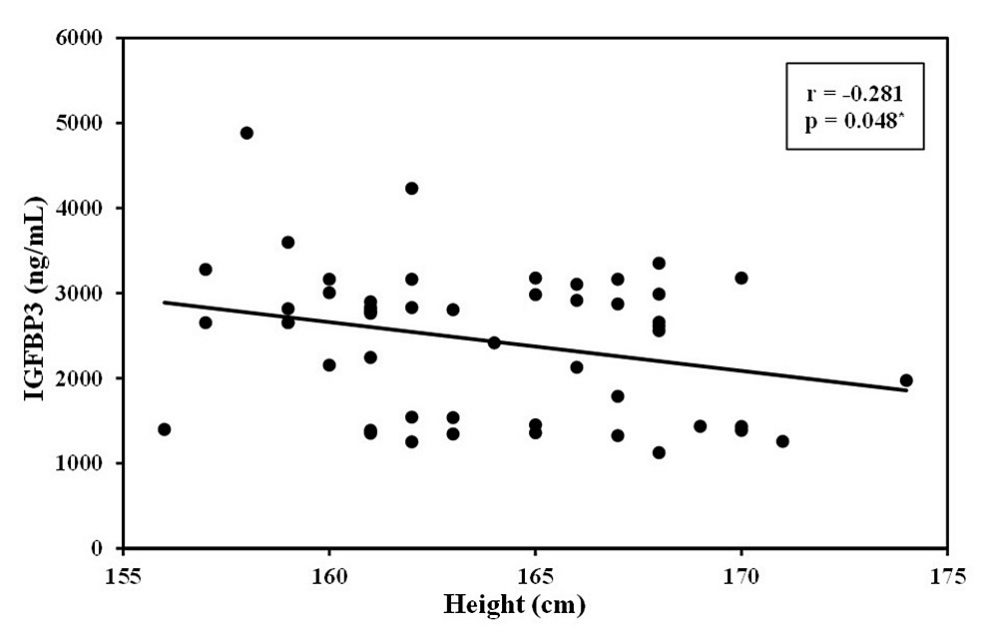
Correlation between IGFBP3 with height in cases group (n = 50) IGFBP3: insulin-like growth factor binding protein 3, r: correlation coefficient, p: probability

**Figure 4:**
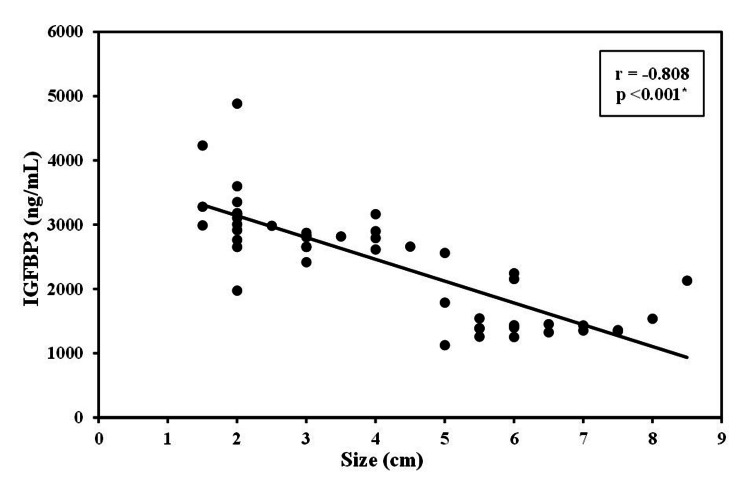
Correlation between IGFBP3 with size in cases group (n = 50) IGFBP3: insulin-like growth factor binding protein 3, r: correlation coefficient, p: probability

The level of IGFBP3 was strongly negatively correlated with the age of patients with CRC (r=−0.897, p<0.01). This significant negative correlation was also detected between IGFBP3 levels and the size of the tumor (r=−0.808, p <0.01). Additionally, the height of the patients was positively correlated with IGF1 (r=0.453, p<0.01) and negatively correlated with the IGFBP3 level (r=−0.281, p<0.01; Figures 1-4).

## Discussion

The IGF system plays a critical role in the pathogenesis of dysplasia and neoplasia [8]. In this study, the levels of IGF1 and IGFBP3 levels were assessed in 50 patients with untreated CRC and compared to 50 age and sex-matched controls. The demographic and the clinicopathological characteristics of the patients in relation to the levels of IGF1 and IGFBP3 were evaluated. We found evidence that increased IGF1 levels are associated with CRC. These results are in accordance with the results obtained by previous studies [16,17] and might suggest that elevated serum IGF1 levels may have a role in CRC development. The current clinical practice for colorectal cancer surveillance in individuals with acromegaly and increased blood IGF1 levels is augmented by our findings. Our results support the already known theory of the possible causal relation between IGF1 (which is elevated in acromegaly) and the high incidence of CRC reported in acromegalic patients. The possible mechanisms explaining the increased colorectal cancer risk might be a direct effect of IGF1 on cell growth or an indirect effect, such as insulin resistance and raised insulin levels [18]. Experimental studies demonstrated that IGF1 and its signaling pathways have a role in colorectal carcinogenesis. The activation of the mitogen-activated protein kinase and phosphoinositide 3-kinase pathways by IGF1 can enhance cell proliferation and inhibition of apoptosis, each of which could contribute to tumor development. IGFI may potentially accelerate cancer progression by inducing the expression of vascular endothelial growth factor, which regulates the formation of new blood vessels [17]. IGF1 receptors are expressed by colonocytes and are typically overexpressed in cancer cells. In colorectal tissue, IGF1 has been demonstrated to increase cellular proliferation, and blocking the IGF1-receptor with a monoclonal antibody decreases cell proliferation [16].

Despite widespread experimental data that IGF1 plays a role in colorectal tumorigenesis, results from previous epidemiological studies have been controversial, with null or weak positive associations found that did not meet the statistical significance threshold [7,16]. Our results show that the average IGF1 was significantly higher among patients with liver metastasis, lymph node spread, and lymphovascular invasions and did not show a significant association with gender, smoking status, family history, or primary site of colorectal cancer. Similar to our results, Ma et al. [17] reported that IGF1 levels are higher among case subjects than among control subjects at each level of IGFBP3, independent of age. This finding raises concerns that long-term treatment with growth hormone as suggested for elderly subjects to slow the effects of aging may be linked to an increased risk of neoplasia. In another case-control study assessing 23 cases, circulating levels of IGF1 were not associated with colorectal cancer [19]. IGF1 gene expression in CRC was not associated with clinicopathological characteristics according to Peters et al., [20] whereas Shiratsuchi et al. [21] reported that IGF1 gene expression in CRC was associated with tumor size, depth of tumor invasion, lymphatic invasion, and lymphovascular invasion. IGF1 bioactivity is regulated in part by IGFBPs, the majority of which are coupled to IGFBP3. Higher levels of IGFBP3 enhance IGF1 serologic binding capability while lowering IGF1 bioavailability in the blood. IGFBP3 has been demonstrated to cause apoptosis and inhibit proliferation in colon cancer cell lines, in addition to its IGF-binding characteristics [16,22]. Our study demonstrated significantly lower levels of IGFBP3 in cases compared to control subjects. Lower IGFBP3 was significantly evident among patients with liver and lymph node metastasis and those with lymphovascular invasion in addition to patients with positive family history. A significant negative correlation was also detected between IGFBP3 levels and the size of the tumor. The negative association in our study for IGFBP3 and colorectal cancer is consistent with its anticipated anti-tumorigenic effects. Epidemiologic studies evaluating the link between IGFBP3, and colorectal cancer have yielded conflicting results, with inverse, positive, and null findings all previously reported [7,19,22-26].

In the current study, the level of IGFBP3 was strongly negatively correlated with the age of patients with CRC. This finding is similar to results obtained in previous studies [17]. It has been postulated that these alterations in the IGFs may play a role in aging-related carcinogenesis. A number of pharmacologic medicines that target the IGF system have been discovered, however, they have yet to be successful in clinical trials treating colon cancer patients. Somatostatin analogues or growth hormone-releasing hormone antagonists may provide a more potent technique for suppressing the growth hormone-IGF1 axis, but the cost and side effects are key considerations. Although circulating IGF1 levels can be modified, it is unclear how long an intervention focused on changing IGF1 concentrations would have to be used for measurable results [16]. Efforts should be directed to develop and implement IGF-targeted therapies for colorectal cancer prevention in susceptible individuals.

Finally, our findings raise concerns that long-term use of growth hormones may increase the incidence of epithelial malignancies. Although such therapy has been proposed to delay some of the effects of aging, any objective benefits of such intervention should be weighed against the potential risk.

One limitation of our study is that all participants' IGF1 levels were assessed just once at baseline, and it is likely that these values do not reflect exposure levels throughout time. However, a recent study [16] discovered an Intraclass Correlation Coefficient (ICC) value of 0.78 between IGF1 measures acquired four years apart in a sample of the population, demonstrating that a single measurement offers a good estimate of IGF1.

## Conclusions

High IGF1 levels and low concentrations of IGFBP3 are related to colorectal cancer and were significantly associated with liver metastasis, lymph node spread, and lymphovenous invasions. Further research is recommended to investigate if circulating IGF1 and IGFBP3 levels can be used to identify people at high risk of colorectal cancer and to explore potential lifestyle or pharmaceutical ways to lower IGF1 bioactivity as a risk reduction strategy targeting high-risk individuals. More studies are needed to determine the role of IGF1 and IGFBP3 in the early detection of CRC and may help as a prognostic factor and for follow-up of patients with CRC.
